# Association of lockdowns with the protective role of ultraviolet-B (UVB) radiation in reducing COVID-19 deaths

**DOI:** 10.1038/s41598-021-01908-w

**Published:** 2021-11-24

**Authors:** Rahul Kalippurayil Moozhipurath, Lennart Kraft

**Affiliations:** Faculty of Economics and Business, Goethe University Frankfurt, Theodor-W.-Adorno-Platz 4, 60629 Frankfurt, Germany

**Keywords:** Applied immunology, Infection, Infectious diseases, Innate immune cells, Innate immunity, Diseases, Endocrinology, Health care, Health occupations, Medical research, Pathogenesis

## Abstract

Nations are imposing unprecedented measures at a large scale to contain the spread of the COVID-19 pandemic. While recent studies show that non-pharmaceutical intervention measures such as lockdowns may have mitigated the spread of COVID-19, those measures also lead to substantial economic and social costs, and might limit exposure to ultraviolet-B radiation (UVB). Emerging observational evidence indicates the protective role of UVB and vitamin D in reducing the severity and mortality of COVID-19 deaths. This observational study empirically outlines the protective roles of lockdown and UVB exposure as measured by the ultraviolet index (UVI). Specifically, we examine whether the severity of lockdown is associated with a reduction in the protective role of UVB exposure. We use a log-linear fixed-effects model on a panel dataset of secondary data of 155 countries from 22 January 2020 until 7 October 2020 (n = 29,327). We use the cumulative number of COVID-19 deaths as the dependent variable and isolate the mitigating influence of lockdown severity on the association between UVI and growth rates of COVID-19 deaths from time-constant country-specific and time-varying country-specific potentially confounding factors. After controlling for time-constant and time-varying factors, we find that a unit increase in UVI and lockdown severity are independently associated with − 0.85 percentage points (p.p) and − 4.7 p.p decline in COVID-19 deaths growth rate, indicating their respective protective roles. The change of UVI over time is typically large (e.g., on average, UVI in New York City increases up to 6 units between January until June), indicating that the protective role of UVI might be substantial. However, the widely utilized and least severe lockdown (governmental recommendation to not leave the house) is associated with the mitigation of the protective role of UVI by 81% (0.76 p.p), which indicates a downside risk associated with its widespread use. We find that lockdown severity and UVI are independently associated with a slowdown in the daily growth rates of cumulative COVID-19 deaths. However, we find evidence that an increase in lockdown severity is associated with significant mitigation in the protective role of UVI in reducing COVID-19 deaths. Our results suggest that lockdowns in conjunction with adequate exposure to UVB radiation might have even reduced the number of COVID-19 deaths more strongly than lockdowns alone. For example, we estimate that there would be 11% fewer deaths on average with sufficient UVB exposure during the period people were recommended not to leave their house. Therefore, our study outlines the importance of considering UVB exposure, especially while implementing lockdowns, and could inspire further clinical studies that may support policy decision-making in countries imposing such measures.

## Introduction

Nations are imposing unprecedented non-pharmaceutical intervention measures at a large scale to contain the extent of COVID-19 pandemic^1,2^. Recent studies indicate that non-pharmaceutical interventions such as lockdowns, ceasing business operations, and closing schools may have substantially slowed down the growth of COVID-19^1–3^, indicating their protective role. Emerging observational evidence on the epidemiology of COVID-19 shows that vitamin D deficiency might be a risk factor for COVID-19 incidence, severity, and deaths^4–7^. They also indicate the protective role of a significant source of vitamin D—ultraviolet-B radiation (UVB)^8^—in mitigating COVID-19 deaths. In addition to substantial economic and social costs, an unintended consequence of lockdown is the likelihood of limited exposure to UVB. However, to the best of our knowledge, no empirical study has explored the association between the severity of lockdown, the subsequent reduction in UVB exposure, and the number of deaths attributed to COVID-19 (COVID-19 deaths).

In this observational study of secondary data, we empirically outline the independent protective roles of lockdown and UVB as measured by ultraviolet index (UVI) and subsequently examine whether the severity of lockdown is associated with a reduction in the protective role of UVB. After controlling for time-constant and time-varying factors, we find that a unit increase in UVI and lockdown severity are independently associated with 0.85 percentage points (p.p) and 4.7 p.p decline in the growth rate of COVID-19 deaths. The change of UVI over time is typically large (e.g., Fig. S1 in Supplementary Appendix on an average UVI in New York City increases up to 6 units between January until June), indicating that the protective role of UVI might be substantial. These declines indicate the protective roles of UVI and lockdowns. Surprisingly, the widely utilized lockdown with the least severity (e.g., recommendation to not leave the house) is already associated with almost complete mitigation of the protective role of UVI by 0.76 p.p, which represents a decline of 81%.

## Association of lockdown severity and UVB radiation with cumulative COVID-19 deaths

In general, non-pharmaceutical interventions such as the lockdowns aim to reduce the likelihood of transmission of the virus by limiting the movement of people, reducing the contact among individuals via restricting economic activities such as closing restaurants^1^. Earlier studies indicate that such large-scale non-pharmaceutical intervention measures might have slowed down COVID-19 spread, indicating their protective role, thereby providing public health benefits^1,3,9^. However, such policies’ unintended health consequences (e.g., reduced exposure to UVB radiation) are largely unknown.

Prior studies indicate that UVB radiation plays a protective role in human health^10–14^. Humans get vitamin D either via diet (natural food, fortified food, or supplements) or skin synthesis by UVB radiation exposure^15^. Likelihood of UVB exposure and subsequent vitamin D synthesis undergo substantial variation according to several time-varying and time-constant factors such as latitude^15^, seasons^15^, time of the day^15^, lifestyle^16,17^, mobility^18^, age^15^, skin pigmentation^15^, and obesity^19^. Prior studies associate vitamin D deficiency with the likelihood of weakened immune response^20–22^, infectious respiratory diseases^15,23,24^, and the severity and mortality^25^.

Early evidence indicates that lockdown severity^2^ and weather factors such as temperature and humidity may reduce the likelihood of transmission of the SARS-CoV-2 virus (Severe Acute Respiratory Syndrome Coronavirus 2), which causes COVID-19^26^. Even though UV radiation may help in reducing the likelihood of transmission by inactivating viruses in fomite transmission^27^, emerging epidemiological evidence related to COVID-19 suggests a protective role of UVB and the plausible role of vitamin D in improving immunity and decreasing the likelihood of COVID-19 severity and mortality^4–8,28^.

Specifically, emerging COVID-19 studies provide evidence of the protective role of UVB as well as vitamin D^8,29–31^. Studies indicate that 1,25-dihydroxy vitamin D [1,25 (OH)_2_D], one of vitamin D’s active forms, modulates innate and adaptive immune systems^4,32^ as well as renin-angiotensin system (RAS)^32–34^. Further, studies note that it may reduce the risk of cytokine storm^4,32^ by modulating the inflammatory response. Furthermore, studies indicate that it plays a role in stimulating antimicrobial peptides with antiviral properties such as defensins and human cathelicidin^4,32,35,36^. Emerging COVID-19 studies also provide evidence of the role of vitamin D deficiency in the incidence^29,30,37,38^, severity^37,39^, and mortality^31,40–42^ associated with COVID-19^43^. A quasi-experimental study notes that vitamin D supplementation is associated with a higher chance for survival in elderly hospitalized patients^44^. Further, a recent randomized placebo-controlled study provides early evidence that vitamin D supplementation may help in SARS-CoV-2 (Severe Acute Respiratory Syndrome Coronavirus 2) viral clearance^45^. Furthermore, a pilot randomized clinical study by Castillo et al.^41^ notes that Calcifediol, a metabolite of vitamin D, seems to mitigate the severity of COVID-19.

**Figure 1:**
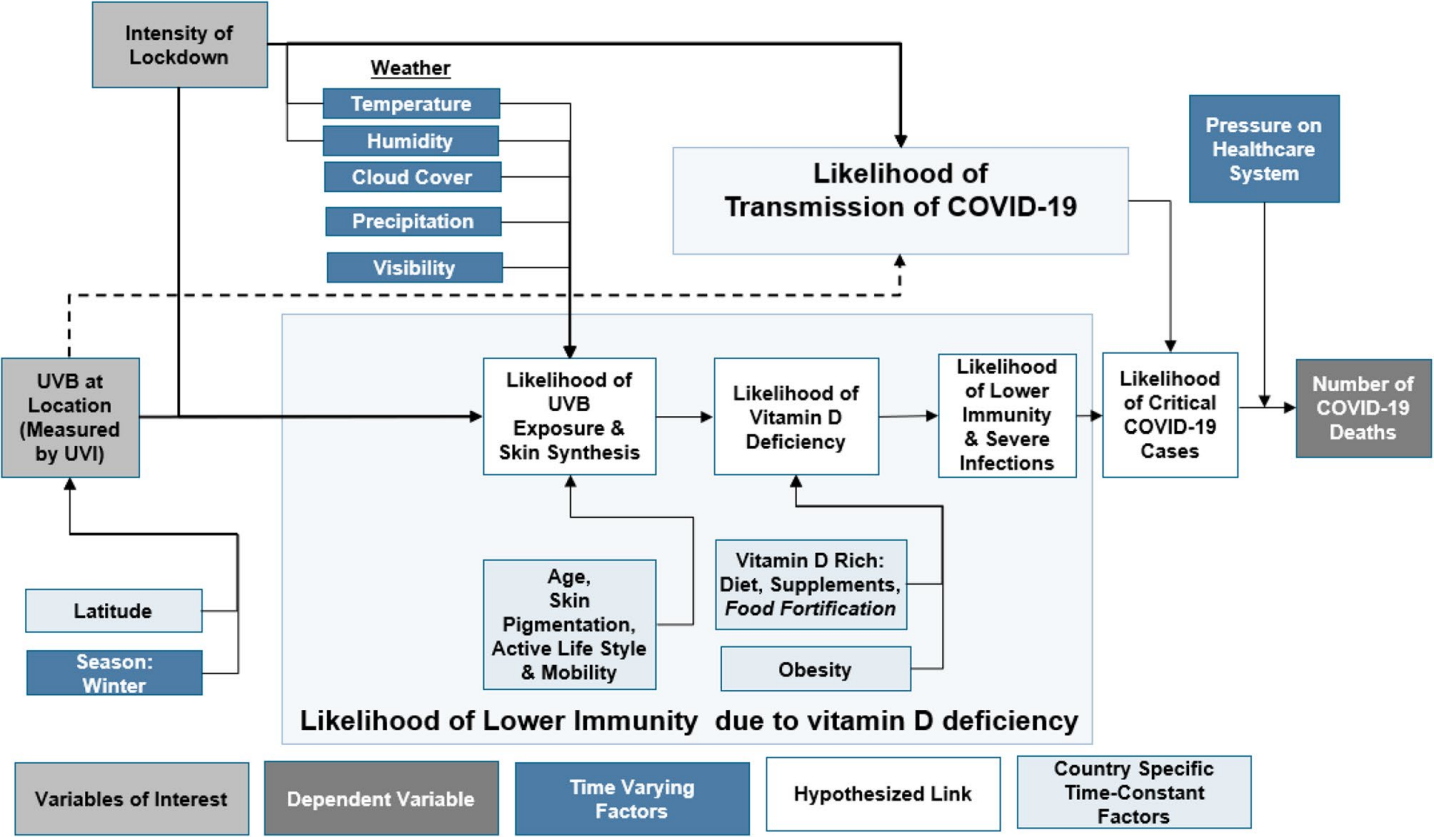
Explanation of the Association of Severity of Lockdown & UVB Radiation with Cumulative COVID-19 Deaths. *Note* We extended the theoretical framework of the protective role of UVB radiation in reducing COVID-19 deaths from Moozhipurath et al.^8^.

We explain the plausible association between the severity of lockdown and the subsequent reduction in the likelihood of UVB exposure with the COVID-19 deaths in Fig. 1. Figure 1 also summarizes various time-varying (e.g., weather, season, etc.) and time-constant factors (age, skin pigmentation, latitude, etc.) affecting the likelihood of UVB exposure and subsequent vitamin D synthesis. In light of the evidence from the emerging studies regarding the role of vitamin D^4–8^, we anticipate that an increased UVB radiation might be associated with a reduction in the number of COVID-19 deaths as it might affect the likelihood of vitamin D deficiency^8^ and the transmission of COVID-19. Although lockdowns might help reduce the likelihood of transmission of SARS-CoV-2 virus^2^, they potentially also reduce the likelihood of UVB radiation exposure, increasing the likelihood of vitamin D deficiency, as indicated in Fig. 1^8^. Therefore, we anticipate that the severity of lockdown and UVB radiation are independently associated with a reduction in the number of COVID-19 deaths. However, an increased lockdown severity might also be associated with a reduction in the protective role of UVI in mitigating COVID-19 deaths.

We extend the theoretical and methodological framework of Moozhipurath et al.^8^ by exploring the protective role of lockdown in the context of UVB exposure. Although Moozhipurath et al.^8^ study the protective role of UVB radiation, it is not yet clear from their results whether the severity of lockdown is associated with a reduction in the protective role of UVB due to reduced likelihood of exposure. We also empirically explore in detail the different types of lockdown severities and whether and which lockdown severity mitigates the protective role of UVI. Thus, the results of this study aim to support the policy decision-making related to COVID-19 in countries that are currently implementing lockdowns or are considering them.

Furthermore, Moozhipurath et al.^8^ investigated the protective role of UVB radiation when the Northern Hemisphere transitioned to spring and Southern Hemisphere countries transitioned to their autumn season. During their study period until 8 May 2020, the COVID-19 outbreak was focused mainly on European countries^8^. Therefore, it is not clear whether their results concerning the protective role of UVB radiation are valid with a longer time horizon in both hemispheres covering different seasons. This study uses secondary data, namely weather data covering 495 days from 1 June 2019 until 7 October 2020, COVID-19 data covering 260 days from 22 January 2020 until 7 October 2020, and lockdown data covering 281 days from 1 January 2020 until 7 October 2020, across 163 countries.

## Data and methods

### Description of data

To empirically estimate the independent protective roles of lockdown, UVB, and the mitigating influence of lockdown severity on the protective role of UVB in reducing COVID-19 deaths, we use the dataset based on secondary data^46^ as outlined in Table 1^8^. The dataset covers 495 days from 1 June 2019 until 7 October 2020 across 163 countries. Out of these 163 countries, 155 countries reported COVID-19 deaths, starting from 22 January 2020, and 155 reported more than 20 COVID-19 infections before 7 October 2020. To ensure that our results are not biased by countries at a very early stage of COVID-19 outbreak, we focus on the above 155 countries^8^ and drop the first 20 daily observations of every country after reporting the first COVID-19 infection^8^.

**Table 1:** Summary of dataset.

Number of countries in the world	195
Number of countries in our dataset	163
… > 0 cumulated COVID-19 deaths before 7 October 2020	155
… > 20 cumulated COVID-19 infections before 7 October 2020	155
Latitude and longitude data source for each state that is used to match weather data	Geocoder (Python)
COVID-19 data source (JHU CSSE COVID-19 Data)	https://github.com/CSSEGISandData/COVID-19^47^
Weather data source	https://darksky.net/
Lockdown severity data source	https://www.bsg.ox.ac.uk/research/research-projects/coronavirus-government-response-tracker^48,49^
Granularity of data	Daily
Covered time-period	COVID-19 data: 22 January 2020–7 October 2020 (260 days) Weather data: 1 June 2019–7 October 2020 (495 days) Lockdown data: 1 January 2020–7 October 2020 (281 days)

The country-level dataset consists of daily observations of cumulative COVID-19 infections and deaths, ultraviolet index (UVI—closely associated with UVB spectrum of solar radiation), and a set of weather factors that we use as control variables—precipitation index, cloud index, ozone level, visibility level, humidity level, as well as minimum and maximum temperature on a daily basis. We merged this dataset with another dataset containing the severity of lockdown enforced by various governments. We describe each dataset in Table 1. We use the interaction of the lockdown severity with UVI to examine whether higher lockdown severity is associated with a reduction in the protective role of UVI in mitigating COVID-19 deaths. Table S3 in the Supplementary Appendix shows the number of observations of countries used in the analysis, whereas Table S4 shows the countries’ latitude and longitude information used in the analysis.

In Table 2, we present the descriptive statistics of the dataset. On 7 October 2020, the average growth rate of COVID-19 deaths per country was 0.76%, and there were on average 6789 cumulative COVID-19 deaths across these 155 countries. The daily average COVID-19 deaths growth rate per country was 3.4% across the duration of the study. The corresponding average for UVI was 7.44, indicating a moderate to high risk of harm from sun exposure. We use cumulative COVID-19 deaths as the primary dependent variable to test our hypothesis that lockdowns mitigate the protective role of UVB radiation. Based on the severity of governmental advice and restrictions, there are four different severity levels that could decrease skin exposure to UVB radiation ranging from “no measures” to “requiring the population not to leave the house with minimal exceptions”. On 7 October 2020, 54 countries had not implemented a lockdown, whereas 56 countries recommended their people not to leave the house. 43 countries required people to not leave the house except for daily exercises, grocery shopping, and essential trips, whereas 2 countries implemented a severe lockdown and required their people not to leave the house with minimal exceptions.

**Table 2:** Descriptive statistics.

Variable	Number of countries	Number of observations	Mean	SD	Min	Max
Cumulated COVID-19 deaths on 7 October	155	155	6789	23,920	1	211,801
Growth rate of cumulative COVID-19 deaths on 7 October	155	155	0.0076	0.016	0	0.13
Daily growth rate of cumulative COVID-19 deaths	155	29,333	0.034	0.13	− 1	9
Time-passed by from first reported infection until 7 October	155	155	219	20	148	260
Daily ultraviolet index (UVI)	155	31,044	7.44	2.91	0	15
Daily precipitation index	155	31,044	0.32	0.32	0	1
Daily cloud index	155	31,044	0.49	0.32	0	1
Daily ozone level	155	31,044	289	33	229	481
Daily visibility level	155	31,044	15.5	1.60	0.12	16.1
Daily humidity level	155	31,044	0.66	0.22	0.02	1
Minimum temperature per day within a country	155	31,044	15.75	8.23	− 31.85	36.76
Maximum temperature per day within a country	155	31,044	26.73	8.89	− 18.17	51.22
Lockdown severity	0	1		2	3	
Description of lockdown severity	No measures	Recommend to not leave the house		Require not to leave the house with exceptions only for daily exercises, grocery shopping, and ‘essential’ trips	Require not to leave the house with minimal exceptions (e.g., allowed to leave only once every few days, or only one person can leave)	
Number of countries with lockdown severity at the end of observational period	54	56		43	2	

We drop the first 20 observations after the first infection in a given country. Therefore, we have 31,044 observations which are less than 76,725 (155 countries × 495 days).

### Summary of method

We apply a log-linear fixed-effects model to estimate the mitigating influence of lockdown severity on the association between UVI and growth rates of COVID-19 deaths^8^. We describe and discuss the log-linear fixed-effect model and the structural equation, which we estimate, in the Supplementary Appendix Section 1, building upon and extending Moozhipurath et al.^8^ as well as Hsiang et al.^1^. We chose this type of model since (i) a log-linear model considers percentage rather than absolute changes, the changes in COVID-19 deaths are more comparable over time across countries^8^, and (ii) the fixed-effect model separates the associations of interest from country-specific time-constant factors that are outlined in Fig. 1^8^. The main model isolates the associations of interest from time-varying linear factors, and we do robustness checks with flexible time-trends as mentioned in the Supplementary Appendix Section 3^8^.

Further, Hsiang et al.^1^ use log-linear fixed effects model to analyze the effect of lockdowns on the COVID-19 pandemic, whereas Moozhipurath et al.^8^ and Carleton et al.^50^ follow this approach to investigate the association of UV with the reduction in COVID-19 growth rates^50^. Furthermore, prior epidemiological studies such as Barecca et al. use a fixed-effects approach to study humidity’s role in influenza by isolating its effect from other weather parameters^51^. Such models are also used in the emerging literature^50^ to estimate climate change’s impact on mortality^52^ and the migration pattern of humans^53^.

To assess the respective protective roles of lockdown and UVI in mitigating the growth rates of COVID-19 deaths and subsequently determine whether and which lockdown severity mitigates the protective role of UVI, we estimate three versions of the log-linear fixed-effects model. Model 1 outlines whether a unit increase in the lockdown severity mitigates the association between UVI and the growth rates of COVID-19. Model 2 and model 3 outline whether a more severe lockdown measure (e.g., LD severity 2 or 3 and LD severity 3, respectively) mitigates this association more strongly than a less severe lockdown (LD severity 1 and LD severity 1 or 2, respectively).

## Results

We present our results in Table 3. After controlling for all time-constant and various time-varying factors^8^, we find that unit increases in UVI and lockdown severity are independently associated with 0.85 percentage points (p.p) and 4.7 p.p decline in COVID-19 deaths growth rate, indicating their respective protective roles. However, we find a significant mitigating influence of lockdown severity on the protective role of UVI in reducing the growth rates of COVID-19 deaths. A unit increase of the lockdown severity weakens the association of UVI in reducing the growth rates of COVID-19 deaths by − 44% (= 0.0037/− 0.0085). This decrease represents the average mitigation of a unit increase of the lockdown severity from 0 to 1, 1 to 2, and 2 to 3.

**Table 3:** Results of log-linear fixed-effects model.

	Model 1	Model 2	Model 3
	COVID-19 deaths	COVID-19 deaths	COVID-19 deaths
**Dependent variables**			
UVI	− 0.0085*** (14.49)	− 0.0094*** (15.53)	− 0.0092*** (14.49)
LD	− 0.047*** (32.95)		
LD × UVI	0.0037*** (15.00)		
LD severity 1		− 0.081*** (25.71)	
LD severity 1 × UVI		0.0076*** (15.04)	
LD severity 2 or 3		− 0.027* (4.14)	
LD severity 2 or 3 × UVI		0.0008 (0.24)	
LD severity 1 or 2			− 0.09*** (30.15)
LD severity 1 or 2 × UVI			0.0081*** (16.94)
LD severity 3			− 0.011 (0.76)
LD severity 3 × UVI			− 0.0004 (0.07)
**Control variables**			
Time trend	Linear	Linear	Linear
Country fixed effects	Yes	Yes	Yes
Precipitation index	Yes	Yes	Yes
Cloud index	Yes	Yes	Yes
Ozone level	Yes	Yes	Yes
Visibility level	Yes	Yes	Yes
Humidity level	Yes	Yes	Yes
Temperature (min and max)	Yes	Yes	Yes
Number of coefficients	61 (+ 155 FE)	73 (+ 155 FE)	73 (+ 155 FE)
Number of observations	29,327	29,327	29,327
Number of countries	155	155	155
R-squared within	17.61%	18.16%	17.92%

*LD* Lockdown severity.

+ : *p* < 0.10; *: *p* < 0.05; **: *p* < 0.01; ***: *p* < 0.001. F-statistic for long-run coefficients in parentheses.

Surprisingly, Model 2 and Model 3 outline that the mitigation effect is mostly associated with lockdown severity of level 1 rather than level 2 or level 3 (stricter lockdowns) as the interaction of lockdown severity of level 2 or 3 with UVI is insignificant. Besides, the lockdown severity of level 1 mitigates the association of UVI and growth rates of COVID-19 deaths by − 81% (0.0076/− 0.0094) and, thus, almost completely mitigates the association. Finally, all models outline the significant negative association of lockdown severity and UVI with growth rates of COVID-19 deaths, indicating their protective roles.

To assess the robustness of our results of the primary model—Model 1—we isolate the mitigating influence of lockdown severity on the association of UVI and growth rates of COVID-19 deaths from time trends in flexible ways. Models 4–9 in Tables S1 and S2 in the Supplementary Appendix isolate our findings from linear, square and exponential time trends, which may be similar across countries or even country-specific. Overall, we find consistent results across different model specifications.

**Figure 2:**
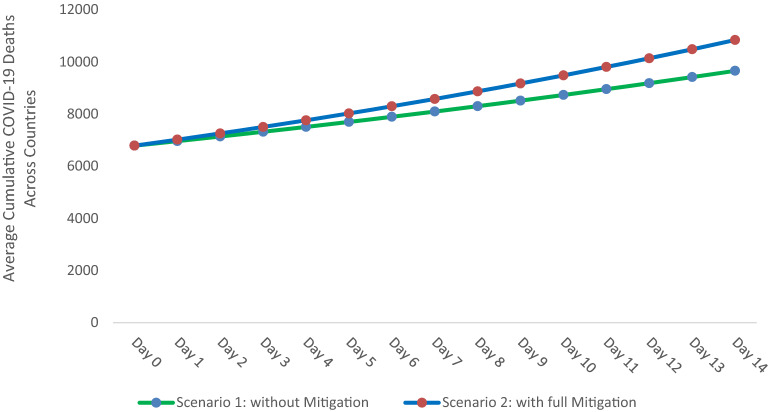
Comparison of “no mitigation of UVI’s protective role by lockdown” versus “full mitigation of UVI’s protective role by lockdown” on cumulative COVID-19 deaths averaged across countries.

Next, we compare two scenarios to illustrate the mitigated protective role of a unit increase of UVI on the cumulative COVID-19 deaths. In scenario 1, UVI’s protective role is not mitigated by lockdown, whereas in scenario 2, UVI’s protective role is fully mitigated. To relate the mitigated protective role with COVID-19 deaths, we take the average number of COVID-19 deaths at the end of the observational period, i.e., 6,789, as cumulative COVID-19 deaths at day 0 shown in Fig. 2. In the full mitigation scenario (Scenario 2), we use the growth rate of COVID-19 deaths in our sample, i.e., 3.4%. Scenario 1, where UVI’s role is not mitigated, uses an average growth rate of 2.55% (i.e., 3.4%—0.85 p.p). Figure 2 outlines that this mitigating influence of lockdown on the protective role of UVI translates into 1,183 or 11% fewer COVID-19 deaths after 14 days.

## Discussion

Our empirical results indicate that large-scale lockdowns are associated with a substantial slowdown in the daily growth rates of COVID-19 deaths consistent with prior studies^1^. However, such measures also significantly reduce the protective role of UVB in COVID-19 deaths.

Specifically, we find that unit increases in UVI and lockdown severity are independently associated with a decline in the growth rate of COVID-19 deaths, indicating their respective protective roles. However, the lowest lockdown severity (recommendation not to leave the house) is already associated with almost complete mitigation of the protective role of UVI in reducing the growth rate of COVID-19 deaths via a reduction of 0.76 percentage points or -81% (*p* < 0.001). Our results are consistent across different model specifications.

Our results suggest that lockdowns in conjunction with adequate exposure to UVB radiation might have provided even more substantial health benefits than lockdowns alone. For example, we estimate that there would be 11% fewer deaths on average with more UVB exposure while people were recommended not to leave their houses.

Our contributions are three-fold. First, to the best of our knowledge, this study is one of the first ones that outlines the association between the severity of lockdown, the subsequent reduction in UVB exposure, and COVID-19 deaths. Second, our study outlines the need for further large-scale clinical studies exploring the role of vitamin D in mitigating the pandemic. Third, even though emerging studies suggest the need for continued large-scale interventions^1,2^, in addition to substantial economic and social costs, the findings of our study indicate that an unintended consequence is the limited UVB exposure, which plausibly increases the risk of COVID-19 deaths. The results of our study can therefore inspire observational or experimental clinical studies that can further support COVID-19 related policy decision-making in countries that are currently implementing lockdowns or are considering them in the future to slow down COVID-19 growth. Specifically, such clinical studies may investigate if sensible sunlight exposure in conjunction with lockdown or with proper social distancing can mitigate COVID-19 deaths. Sensible UVB exposure is possible during lockdown by spending time outside in a garden, on balconies, or by exposing to sunlight through open windows. Further, the results of large scale clinical studies could help guide nations to create awareness regarding the importance of sensible sunlight exposure and also to assist vulnerable populations at a higher risk of vitamin D deficiency—e.g., darker-skinned people living in high latitudes, people with limited mobility or indoor lifestyle (nursing home residents), and vegetarians^8^.

## Limitations

We follow a macro-level statistical backward-looking approach that captures real-life behavior without making any specific assumptions regarding epidemiological parameters^1^. Although this macro-level approach is a crucial strength of the study, the results cannot be interpreted as health guidance, which often comes from clinical studies^1^. Therefore, further clinical studies are needed to establish a causal relationship between UVB-induced vitamin D and COVID-19 deaths.

We use a fixed-effects model that isolates the effect of relevant weather parameters from country-specific time-constant factors^8^. Such country-specific time-constant factors consist of various economic, social, and health factors that are likely to remain relatively stable over the period of our study^8^. The time constant factors include the location (e.g., latitude and longitude), demographics, age composition, gender, genetics, and culture at a country level^8^. More importantly, such time-constant factors include factors that are closely associated with the severity of COVID-19, such as age, gender, mobility, lifestyle of the population, the prevalence of co-morbidities (e.g., obesity, hypertension, etc.), and skin pigmentation^8^. Fixed-effects also may capture factors associated with regular habits such as regular dietary patterns, the proportion of vegetarians in the population, regular dietary supplement consumption, and food fortification that may affect COVID-19 severity^8^.

Our methodology also flexibly controls for various time-varying factors^8^. First, our methods control for relevant confounding time-varying weather factors, including air pollution. Second, we control for various remaining time-varying factors by incorporating linear, quadratic, and exponential time-trends at a country level^8^ in the robustness checks of the Supplementary Appendix. These flexible time-trends control for time-varying factors such as pressure on the health care system and exponential-shaped or s-shaped trends associated with the COVID-19 growth rate.

Although our methodology controls for all time-constant and various time-varying confounding factors, we acknowledge that our method has limitations. First, our method may be limited in capturing some of the time-varying confounding factors that may confound the results. For instance, our methodology may not capture behavioral changes of the people that are likely to be associated with seasonal changes (UVB variation), vitamin D levels, and COVID-19 deaths. For example, such time-varying behaviors include seasonal travel patterns, seasonal nutritional supplement intake, and seasonal dietary habits^8^. Second, although we use governmental measures, we do not have granular data on whether people comply with these measures. Therefore, we have limited information about whether people followed governmental instructions and stayed indoors during the lockdown. Third, we do not have data on the vitamin D level at a population level across these countries corresponding to the UVB radiation that prevents us from directly analyzing the association between COVID-19 deaths and vitamin D levels. Finally, our study is an ecological study based on country-level data and therefore has inherent limitations that are commonly associated with such ecological studies.

Even though we anticipate that reduced likelihood of skin synthesis due to lockdown plausibly explain these associations, we may not be able to rule out the possibility of other UVB-induced mediators—such as nitric oxide^8,10,54^. While we acknowledge there may be confounding factors posing challenges to our analysis, we used statistical methods to account for all time-constant and various time-varying factors as much as possible. We also acknowledge that UVB exposure may not substantially increase the vitamin D synthesis of specific categories of people, such as those wearing cultural protective clothing due to lower exposure to sunlight^15^ and those who are elderly due to less efficient skin synthesis^55^. Although we do not model these factors explicitly, our fixed-effects model accounts for most of these time-constant confounding factors.

## Supplementary Information

Supplementary Information.

## Data Availability

The data used in the study are from publicly available sources. Data regarding COVID-19 are obtained on 9 October 2020 from *COVID-19 Data Repository* by the *Center for Systems Science and Engineering (CSSE)* at *Johns Hopkins University* and can be accessed at https://github.com/CSSEGISandData/COVID-19. Data regarding weather is obtained from *Dark Sky* on the 9 October 2020 and can be accessed at https://darksky.net/. Data regarding lockdown severity is obtained from https://www.bsg.ox.ac.uk/research/research-projects/coronavirus-government-response-tracker. We will make specific dataset used in this study available for any future research. Interested researchers can contact one of the authors via email to get access to the data.
