# Supplementary material for: Association of lockdowns with the protective role of ultraviolet-B (UVB) radiation in reducing COVID-19 deaths
Source: Sci Rep. 2021 Nov 24;11:22851. doi: 10.1038/s41598-021-01908-w (PMC8613284; doi:10.1038/s41598-021-01908-w)
Supplement: Supplementary file 1 — Supplementary Information. [file 41598_2021_1908_MOESM1_ESM.pdf]

Association of Lockdowns with the Protective Role of Ultraviolet-B (UVB) Radiation in Reducing COVID-19 Deaths

Supplementary Appendix

02. October, 2021

Rahul Kalippurayil Moozhipurath*[1], Lennart Kraft[2]

(Faculty of Economics and Business, Goethe University Frankfurt[1,2])

[1]Rahul Kalippurayil Moozhipurath (was affiliated to Goethe University at the time of first submission, when majority of work has been carried out); [2]Lennart Kraft; Faculty of Economics and Business, Goethe University Frankfurt, Theodor-W.-Adorno-Platz 4, 60629 Frankfurt, Germany; email: rahulkm85@gmail.com , Phone: +49-152-1301-0589; email: lennart.kraft@wiwi.uni-frankfurt.de.

Table of Contents

1 Description of Methodology 3
2 Model Selection 7
3 Robustness Checks 7
4 References 13

1 Description of Methodology

In this section, we describe the methodology of our study in more detail. We use a fixed-effect log-linear regression model to estimate the mitigating influence of lockdown severity on the association between UVI and growth rates of COVID-19 deaths . The model builds upon the theoretical framework as represented in Figure 1 in the *Manuscript*. This fixed-effect model is closely related to Moozhipurath, Kraft, and Skiera (2020)[1] as well as Hsiang et al. (2020)[2].

Since a log-linear model considers percentage rather than absolute changes, the changes in COVID-19 deaths are more comparable over time across countries[1]. The fixed-effect model separates the associations of interest from country-specific time-constant factors that are outlined in Figure 1 in the Manuscript[1]. Such time-constant factors may consist of location (e.g., latitude), fixed diet patterns (e.g., intake of supplements or food which is fortified, the proportion of vegetarians, fish eaters, meat eaters, etc. in a population) as well as socio-economic factors which may be stable over our study period[1]. Further, the fixed-effect model may also capture time-constant factors directly related to vitamin D synthesis, such as age, skin pigmentation, among others[1]. Additionally, we include time-varying variables such as weather factors that may affect the likelihood of exposure to UVB radiation and subsequent vitamin D synthesis such as temperature, precipitation, etc[1]. Furthermore, we include linear and non-linear time-trends to control for further unobservable time-varying confounders[1].

The major difference to Moozhipurath, Kraft, and Skiera (2020)[1] is that this study explores the lockdown's mitigating role on the protective effect of UVB exposure in reducing COVID-19 deaths. Therefore, this study extends the methodology described by Moozhipurath, Kraft, and Skiera (2020)[1] by focusing on the two additional sets of variables related to lockdown severity. Those additional sets consist of (i) variables representing the

lockdown severity and (ii) variables representing the interaction of the lockdown severity and UVI. Thus, we use the following model to explain the number of COVID-19 deaths:

$$D_{i,t} = D_{i,t-1} \times e^{\gamma + \sum_{j=0}^{5}[UVI_{i,t-j}\beta_{UVI,j} + LD_{i,t-j}\beta_{LD,j} + UVI \cdot LD_{i,t-j}\beta_{UVI \cdot LD,j} + C_{i,t-j}\beta_{C,j}] + FI_{i,t}\beta_{FI} + u_i + \epsilon_{i,t}} \quad (1)$$

Similar to Moozhipurath, Kraft, and Skiera (2020)[1], $D_{i,t}$ represents the cumulative COVID-19 deaths for a country i at time point t (in days). $D_{i,t}$ relates to the explanatory variables via an exponential growth model[1] on the right-hand side of the equation (1). As outlined by Moozhipurath, Kraft, and Skiera (2020)[1], our exponential growth model also flexibly allows for different shapes for the cumulative COVID-19 deaths.

In this study, we use an exponential growth model that consists of eight explanatory parts, extending the model of Moozhipurath, Kraft, and Skiera (2020)[1], which has six explanatory parts.

1) γ represents the daily growth rate of COVID-19 deaths from $D_{i,t-1}$ to $D_{i,t}$ that is independent of the factors presented in Figure 1 as shown in the *Manuscript*[1] similar to Moozhipurath, Kraft, and Skiera (2020)[1]. γ covers attributes specific to virus and its transmission such as its basic reproductive rate R_0 combined with its lethality[1].
2) $UVI_{i,t-j}$ represents the daily UVI for a country i on a day t, lagged by j weeks. Therefore, similar to Moozhipurath, Kraft, and Skiera (2020)[1], $\beta_{UVI,j}$ reflects the effect of UVI lagged by j weeks[1].
3) $LD_{i,t-j}$ represents the lockdown severity LD for a country i at day t lagged by j weeks. $\beta_{LD,j}$ reflects the effect of LD lagged by j weeks. LD can either consist of one variable lagged by j weeks as in Model 1, or it can consist of 2 dummy variables as in model 2 or 3. The first dummy variable of model 2 is equal to one if the lockdown severity is at least equal to 1. In contrast, the second dummy variable of model 2 is

equal to one if the lockdown severity is at least equal to two. The first dummy variable of model 3 is equal to one if the lockdown severity is at least equal to 1. In contrast, the second dummy variable of model 3 is equal to one if the lockdown severity is equal to three. Similarly, $\beta_{LD,j}$ can measure the effect of a unit increase in the lockdown severity as in model 1. $\beta_{LD,j}$ can also measure the effect of a specific lockdown severity as in model 2 and model 3.

4) $UVI \cdot LD_{i,t-j}$ represents the interaction of $UVI_{i,t-j}$ and the lockdown severity $LD_{i,t-j}$ for a country i at day t lagged by j weeks. $\beta_{UVI \cdot LD,j}$ reflects the effect of this interaction lagged by j weeks. We either interact UVI with variable LD for model 1 or we interact UVI with the dummy variable for model 2 and model 3. Therefore, $\beta_{UVI \cdot LD,j}$ reflects the effect of a unit increase of UVI lagged by j weeks if the lockdown severity increases by one unit as for model 1. For model 2, $\beta_{UVI \cdot LD,j}$ can represent the change of the effect of a unit increase of UVI when lockdown severity is 1 as compared to when lockdown severity is 0. $\beta_{UVI \cdot LD,j}$ can also represent the change of the effect of a unit increase of UVI when lockdown severity is 2 or 3 as compared to when lockdown severity is 1. For model 3, $\beta_{UVI \cdot LD,j}$ can represent the change of the effect of a unit increase of UVI when lockdown severity is 1 or 2 as compared to when lockdown severity is 0. $\beta_{UVI \cdot LD,j}$ can also represent the change of the effect of a unit increase of UVI when lockdown severity is 3 as compared to when lockdown severity is 1 or 2.

5) $C_{i,t-j}$ stands for the set of control variables, similar to Moozhipurath, Kraft, and Skiera (2020)[1] . These control variables consists of weather factors such as cloud index, ozone level, precipitation index, visibility level (an indicator of air pollution), humidity level, as well as temperature (minimum and maximum) for a country i on

day t , lagged by j weeks[1]. The vector $\beta_{C,j}$ identifies the effect of these control variables lagged by j weeks[1].

6) $FI_{i,t}$ represents the time passed by since the COVID-19 infection is first reported in a country i on day t and β_{FI} identifies the associated effect[1], similar to Moozhipurath, Kraft, and Skiera (2020)[1].
7) u_i represents all time-constant country-specific factors impacting the growth rate of cumulative daily COVID-19 deaths (e.g., time-constant country-specific factors – e.g., population factors such as demographics, age composition, gender, culture, location, lifestyle, mobility, and co-morbidities as well as diet-related factors such as consumption of dietary supplements, diet rich in vitamin D (fish, meat and egg) and food fortification, similar to Moozhipurath, Kraft, and Skiera (2020)[1].
8) $\epsilon_{i,t}$ represents all remaining factors that are not identified, but have an effect on the cumulative daily COVID-19 deaths (i.e., all non-linear differences of growth rates with respect to time as well as country-specific linear differences of growth rates with respect to time), similar to Moozhipurath, Kraft, and Skiera (2020)[1]. They could also result from a decreasing number of people who could potentially become infected or contagious due to immunity, lockdowns in a country over time, mutation or changes in the virus in a specific country over time, systematic false reports of the dependent variable[1].

An appropriate transformation as outlined in Moozhipurath, Kraft, and Skiera (2020)[1] results in the following estimable equation.

$$\Delta \ln(\ddot{D}_{i,t}) = \sum_{j=0}^{5}\left[\ddot{UVI}_{i,t-j}\beta_{UVI,j} + \ddot{LD}_{i,t-j}\beta_{LD,j} + \ddot{UVI \cdot LD}_{i,t-j}\beta_{UVI\cdot LD,j} + \ddot{C}_{i,t-j}\beta_{C,j}\right] + \ddot{FI}_{i,t}\beta_{FI} + \ddot{\epsilon}_{i,t} \quad (2)$$

γ and u_i do not appear in the equation anymore and a linear regression can identify all other coefficients as outlined in Moozhipurath, Kraft, and Skiera (2020)[1]. The sum of the

coefficients β for each variable measures the long-term effect of a permanent unit change of the respective variable and outlines by how many percentage points the growth-rate of COVID-19 deaths changes. If $\sum_{j=0}^{5} \beta_{UVI,j} = -0.085$, then a permanent unit increase of UVI is associates with a 0.85 percentage points decline in the growth rates of COVID-19 deaths.

Equation (2) also shows why we can only use those observations where cumulative COVID-19 deaths are larger than zero. This condition explains the difference between the 31,044 observations of 155 countries in Table 2 and the 29,327 observations of 155 countries in Table 3. We listed the number of observations per country we used in the analysis in Table S3.

2 Model Selection

We estimated different versions of our model which varied in the number of weekly lags. We decided to choose a model with 5 weekly lags because we could not find any major differences concerning the estimated coefficients when we increased the number of lags. We favored a more parsimonious model.

3 Robustness Checks

To assess the robustness of our results of the primary model - Model 1 - we isolate the mitigating influence of lockdown severity on the association of UVI and growth rates of COVID-19 deaths from time trends in flexible ways. Models 4 – 9 in Table S1 and Table S2 isolate our findings from linear, square, and exponential time trends, which may be similar across countries or even country-specific. Overall, we find consistent results across different model specifications. A permanent unit increase of UVI and a permanent unit increase in lockdown severity are independently associated with a -0.35 – -0.94 and -4.4 – -6.6 percentage points reduction in growth rates of COVID-19 deaths respectively, according to different model specifications. Results also indicate that a permanent unit increase in

lockdown severity weakens the association of UVI in reducing the growth rates of COVID-19 deaths by 0.22 – 0.45 percentage points. Table S4 shows the latitude and longitude information of the countries used in the analysis.

Table S1: Robustness Checks with Fixed-Effects Model

	Model 4	Model 5	Model 6
Dependent Variables	COVID-19 Deaths	COVID-19 Deaths	COVID-19 Deaths
UVI	-0.0084*** (15.40)	-0.0094*** (13.81)	-0.0035* (3.98)
LD	-0.044*** (29.97)	-0.066*** (44.85)	-0.048*** (35.97)
LD x UVI	0.0036*** (14.34)	0.0045*** (16.34)	0.0022* (5.87)
**Control Variables**			
Time Trend	Linear and Square	Country-specific Linear	Country-specific Linear and Square
Country Fixed Effects	Yes	Yes	Yes
Precipitation index	Yes	Yes	Yes
Cloud index	Yes	Yes	Yes
Ozone level	Yes	Yes	Yes
Visibility Level	Yes	Yes	Yes
Humidity level	Yes	Yes	Yes
Temperature (min and max)	Yes	Yes	Yes
Number of Coefficients	62 (+ 155 FE)	60 (+ 155 FE + 155 TCSE)	60 (+ 155 FE + 310 TCSE)
Number of Observations	29,327	29,327	29,327
Number of Countries	155	155	155
R-squared Within	18.44%	22.31%	27.44%

Note: +: p < 0.10, *: p < 0.05, **: p < 0.01. F-statistic for long-run coefficient in parentheses. TCSE stands for time country-specific effects. LD stands for Lockdown Severity.

Table S2: Robustness Checks with Fixed-Effects Model

	Model 7	Model 8	Model 9
Dependent Variables	COVID-19 Deaths	COVID-19 Deaths	COVID-19 Deaths
UVI	-0.0085*** (14.54)	-0.0085*** (14.38)	-0.0035* (3.92)
LD	-0.047*** (33.01)	-0.047*** (32.43)	-0.048*** (36.36)
LD x UVI	0.0037** (15.01)	0.0037*** (14.59)	0.0022* (5.90)
**Control Variables**			
Time Trend	Linear and Exponential	Linear and Country-specific Exponential	Country-specific Linear, Square and Exponential
Country Fixed Effects	Yes	Yes	Yes
Precipitation index	Yes	Yes	Yes
Cloud index	Yes	Yes	Yes
Ozone level	Yes	Yes	Yes
Visibility Level	Yes	Yes	Yes
Humidity level	Yes	Yes	Yes
Temperature (min and max)	Yes	Yes	Yes
Number of Coefficients	62 (+ 155 FE)	61 (+ 155 FE + 155 TSCE)	60 (+ 155 FE + 465 TSCE)
Number of Observations	29,327	29,327	29,327
Number of Countries	155	155	155
R-squared Within	17.61%	17.72%	27.52%

Note: +: p < 0.10, *: p < 0.05, **: p < 0.01. F-statistic for long-run coefficient in parentheses. TCSE stands for time country-specific effects. LD stands for Lockdown Severity.

Figure S1: Average Ultraviolet-Index (UVI) of New York City, NY[3]

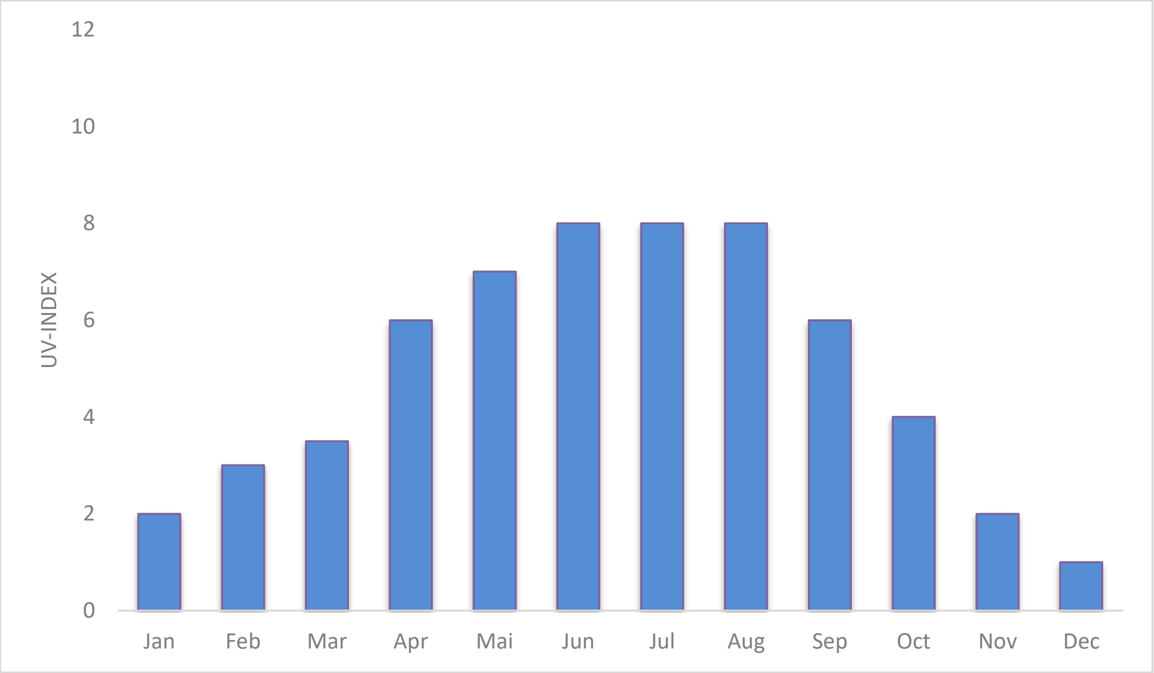

Table S3: Number of Observations (Obs,) of Countries Used in Analysis

Country	Obs.	Country	Obs.	Country	Obs.	Country	Obs.
Afghanistan	197	Denmark	205	Kuwait	186	Rwanda	130
Albania	192	Djibouti	180	Kyrgyzstan	185	San Marino	205
Algeria	207	Dominican Republic	202	Latvia	187	Saudi Arabia	197
Andorra	199	Ecuador	202	Lebanon	211	Senegal	189
Angola	183	Egypt	213	Lesotho	90	Serbia	197
Argentina	200	El Salvador	184	Liberia	186	Sierra Leone	167
Australia	220	Estonia	196	Libya	179	Singapore	200
Austria	207	Eswatini	174	Lithuania	200	Slovakia	189
Azerbaijan	202	Ethiopia	185	Luxembourg	203	Slovenia	198
Bahrain	205	Fiji	68	Madagascar	143	Somalia	182
Bangladesh	195	Finland	200	Malawi	170	South Africa	194
Barbados	185	France	235	Malaysia	204	South Sudan	145
Belarus	190	Gabon	189	Mali	178	Spain	218
Belgium	210	Gambia	186	Mauritania	189	Sri Lanka	193
Belize	180	Georgia	186	Mauritius	185	Sudan	190
Benin	184	Germany	212	Mexico	202	Suriname	187
Bolivia	192	Ghana	189	Moldova	195	Sweden	211
Bosnia and Herzegovina	198	Greece	206	Morocco	201	Switzerland	207
Botswana	173	Guatemala	189	Mozambique	135	Syria	181
Brazil	204	Guinea	175	Namibia	89	Taiwan*	234
Brunei	193	Guyana	191	Nepal	144	Tajikistan	142
Bulgaria	189	Haiti	183	Netherlands	205	Tanzania	187
Burkina Faso	193	Honduras	192	New Zealand	192	Thailand	220
Burundi	172	Hungary	199	Nicaragua	184	Togo	194
Cameroon	196	Iceland	202	Niger	182	Trinidad and Tobago	189
Canada	212	India	210	Nigeria	198	Tunisia	199
Central African Republic	137	Indonesia	201	Norway	206	Turkey	192
Chad	162	Iran	213	Oman	190	US	221
Chile	199	Iraq	208	Pakistan	203	Uganda	75
China	241	Ireland	203	Panama	193	Ukraine	200
Colombia	197	Israel	200	Papua New Guinea	71	United Arab Emirates	201
Congo (Kinshasa)	192	Italy	229	Paraguay	195	United Kingdom	215
Costa Rica	197	Jamaica	192	Peru	197	Uruguay	190
Cote d'Ivoire	192	Japan	237	Philippines	233	Uzbekistan	188
Croatia	202	Jordan	194	Poland	199	Venezuela	189
Cuba	191	Kazakhstan	190	Portugal	201	Vietnam	68
Cyprus	194	Kenya	190	Qatar	193	Yemen	160
Czechia	199	Korea, South	230	Romania	199	Zambia	185
		Kosovo	189	Russia	202	Zimbabwe	183
Total Number of Observations				29,327			

Table S4: Latitude (Lat.) and longitude (Long.) of countries used in analysis

Country	Lat.	Long.	Country	Lat.	Long.	Country	Lat.	Long.
Afghanistan	33.94	67.71	Gambia	13.44	-15.31	Norway	60.47	8.47
Albania	41.15	20.17	Georgia	42.32	43.36	Oman	21.51	55.92
Algeria	28.03	1.66	Germany	51.17	10.45	Pakistan	30.38	69.35
Andorra	42.51	1.52	Ghana	7.95	-1.02	Panama	8.54	-80.78
Angola	-11.20	17.87	Greece	39.07	21.82	Papua New Guinea	-6.31	143.96
Argentina	-38.42	-63.62	Guatemala	15.78	-90.23	Paraguay	-23.44	-58.44
Australia	-32.11	141.36	Guinea	9.95	-9.70	Peru	-9.19	-75.02
Austria	47.52	14.55	Guyana	4.86	-58.93	Philippines	12.88	121.77
Azerbaijan	40.14	47.58	Haiti	18.97	-72.29	Poland	51.92	19.15
Bahrain	26.03	50.55	Honduras	15.20	-86.24	Portugal	39.40	-8.22
Bangladesh	23.69	90.36	Hungary	47.16	19.50	Qatar	25.35	51.18
Barbados	13.19	-59.54	Iceland	64.96	-19.02	Romania	45.94	24.97
Belarus	53.71	27.95	India	20.59	78.96	Russia	61.52	105.32
Belgium	50.83	4.47	Indonesia	-0.79	113.92	Rwanda	-1.94	29.87
Belize	17.19	-88.50	Iran	32.43	53.69	San Marino	43.94	12.46
Benin	9.31	2.32	Iraq	33.22	43.68	Saudi Arabia	23.89	45.08
Bolivia	-16.29	-63.59	Ireland	53.14	-7.69	Senegal	14.50	-14.45
Bosnia and Herzegovina	43.92	17.68	Israel	31.05	34.85	Serbia	44.02	21.01
Botswana	-22.33	24.68	Italy	41.87	12.57	Sierra Leone	8.46	-11.78
Brazil	-14.24	-51.93	Jamaica	18.11	-77.30	Singapore	1.28	103.83
Brunei	4.54	114.73	Japan	36.20	138.25	Slovakia	48.67	19.70
Bulgaria	42.73	25.49	Jordan	31.24	36.51	Slovenia	46.15	15.00
Burkina Faso	12.24	-1.56	Kazakhstan	48.02	66.92	Somalia	5.15	46.20
Burundi	-3.37	29.92	Kenya	-0.02	37.91	South Africa	-30.56	22.94
Cameroon	3.85	11.50	Korea, South	35.91	127.77	South Sudan	6.88	31.31
Canada	52.82	-91.70	Kosovo	42.60	20.90	Spain	40.46	-3.75
Central African Republic	6.61	20.94	Kuwait	29.31	47.48	Sri Lanka	7.87	80.77
Chad	15.45	18.73	Kyrgyzstan	41.20	74.77	Sudan	12.86	30.22
Chile	-35.68	-71.54	Latvia	56.88	24.60	Suriname	3.92	-56.03
China	32.89	111.79	Lebanon	33.85	35.86	Sweden	60.13	18.64
Colombia	4.57	-74.30	Lesotho	-29.61	28.23	Switzerland	46.82	8.23
Congo (Kinshasa)	-4.04	21.76	Liberia	6.43	-9.43	Syria	34.80	39.00
Costa Rica	9.75	-83.75	Libya	26.34	17.23	Taiwan*	23.70	121.00
Cote d'Ivoire	7.54	-5.55	Lithuania	55.17	23.88	Tajikistan	38.86	71.28
Croatia	45.10	15.20	Luxembourg	49.82	6.13	Tanzania	-6.37	34.89
Cuba	21.52	-77.78	Madagascar	-18.77	46.87	Thailand	15.87	100.99
Cyprus	35.13	33.43	Malawi	-13.25	34.30	Togo	8.62	0.82
Czechia	49.82	15.47	Malaysia	4.21	101.98	Trinidad and Tobago	10.69	-61.22
Denmark	63.29	-13.34	Mali	17.57	-4.00	Tunisia	33.89	9.54
Djibouti	11.83	42.59	Mauritania	21.01	-10.94	Turkey	38.96	35.24
Dominican Republic	18.74	-70.16	Mauritius	-20.35	57.55	US	40.00	-100.00
Ecuador	-1.83	-78.18	Mexico	23.63	-102.55	Uganda	1.37	32.29
Egypt	26.82	30.80	Moldova	47.41	28.37	Ukraine	48.38	31.17

El Salvador	13.79	-88.90	Morocco	31.79	-7.09	United Arab Emirates	23.42	53.85
Estonia	58.60	25.01	Mozambique	-18.67	35.53	United Kingdom	24.55	-43.90
Eswatini	-26.52	31.47	Namibia	-22.96	18.49	Uruguay	-32.52	-55.77
Ethiopia	9.15	40.49	Nepal	28.17	84.25	Uzbekistan	41.38	64.59
Fiji	-17.71	178.07	Netherlands	21.41	-52.99	Venezuela	6.42	-66.59
Finland	61.92	25.75	New Zealand	-40.90	174.89	Vietnam	14.06	108.28
France	8.31	5.46	Nicaragua	12.87	-85.21	Yemen	15.55	48.52
Gabon	-0.80	11.61	Niger	17.61	8.08	Zambia	-13.13	27.85
			Nigeria	9.08	8.68	Zimbabwe	-19.02	29.15

4 References

1. Moozhipurath, R. K., Kraft, L. & Skiera, B. Evidence of protective role of ultraviolet-B (UVB) radiation in reducing COVID-19 deaths. *Scientific Reports* **10**, 17705 (2020).
2. Hsiang, S. *et al.* The effect of large-scale anti-contagion policies on the COVID-19 pandemic. *Nature* **584**, 262–267 (2020).
3. New York City, NY - Detaillierte Klimaangaben und Monatliche Wettervorhersage. *Weather Atlas* https://www.weather-us.com/de/new-york-usa/new-york-city-klima (2020).
